# Health Motivation as a Predictor of mHealth Engagement Across BMI: Cross-Sectional Survey

**DOI:** 10.2196/71625

**Published:** 2025-12-01

**Authors:** Shao-Hsuan Chang, Lung-Kun Yeh, Daishi Chen, Kae-Kuen Hu, Mei-Ching Yu, Ching-Mao Chang

**Affiliations:** 1 Department of Biomedical Engineering Chang Gung University Taoyuan Taiwan; 2 Department of Ophthalmology Linkou Chang Gung Memorial Hospital Taoyuan Taiwan; 3 College of Medicine Chang Gung University Taoyuan Taiwan; 4 Department of Otolaryngology Shenzhen People’s Hospital Shenzhen China; 5 Professional Master’s Program of Biotechnology Management School of Professional Education and Continuing Studies National Taiwan University Taipei Taiwan; 6 Department of Pediatric Nephrology Linkou Chang Gung Memorial Hospital Taoyuan Taiwan; 7 Center for Traditional Medicine Taipei Veterans General Hospital Taipei Taiwan; 8 School of Chinese Medicine and Institute of Traditional Medicine National Yang Ming Chiao Tung University Taipei Taiwan

**Keywords:** digital health, BMI, health motivation, mobile health, self-efficacy, user engagement, Mandarin-speaking population

## Abstract

**Background:**

Digital health tools, such as mobile apps and wearable devices, have been widely adopted to support self-management of health behaviors. However, user engagement remains inconsistent, particularly among populations with varying BMI. While digital health technologies have the potential to promote healthier behaviors, little is known about how psychological and behavioral factors interact with BMI to influence use patterns.

**Objective:**

This study aimed to explore the relationship between BMI and digital health technology use and to examine how factors such as health awareness, self-efficacy, and health motivation contribute to technology engagement.

**Methods:**

A cross-sectional online survey was conducted from January 2024 to April 2024. A total of 184 valid questionnaire participants were included in this study. The questionnaire was measured on a 5-point Likert scale. Descriptive statistics, chi-square tests, and multiple regression analyses were applied.

**Results:**

Of the participants, 38.6% (71/184) had a BMI<24 kg/m^2^, 42.4% (78/184) had a BMI between 24 and 29.9 kg/m^2^, and 19% (35/184) had a BMI≥30 kg/m^2^. Significant BMI differences were observed based on sex (*P*<.001) and age (*P*<.001) but not based on prior digital health tool use. Use rates for Bluetooth or Wi-Fi devices, wearables, and mobile apps were 32.1% (59/184), 38.6% (71/184), and 39.1% (72/184), respectively. A negative correlation between BMI and mobile app use frequency was identified (*P*=.02). Multiple regression analysis indicated that health motivation significantly predicted digital health use (*P*<.001), whereas health awareness, lifestyle, and self-efficacy did not.

**Conclusions:**

Individuals with higher BMI reported a lower frequency of digital health tool use, potentially due to lower health motivation in the studied population. Health motivation was the strongest predictor of digital health engagement. Integrating personalized medical records into apps may enhance health motivation, thereby improving user engagement and promoting healthier behaviors in individuals with higher BMI.

## Introduction

The digital era has brought an explosion of health-related data from clinical databases, social networks, wearables, and connected medical devices. Among these digital health innovations, mobile health (mHealth) apps and wearable devices stand out, with an expected 3.7 billion downloads globally by consumers and health care professionals [1]. The increasing adoption of digital health is driven by patient convenience, technological advancements, and persistent health care concerns. The transition from traditional to digital and information-based medicine has contributed to the rise of personalized health care, enabling a broader population to engage in health management. This includes those who may not have a formal diagnosis but experience early symptoms or notice early warning signs, often referred to as subhealth status [2]. Sharing health information through digital devices has given rise to a new form of algorithmic surveillance. These technologies now play an increasing role in the medicalization of health. Researchers have proposed different frameworks to explain the medicalization of everyday life through digital technologies, such as quantification, where individuals track health data using devices, and gamification, where playful elements such as points or badges are incorporated to promote user motivation and adherence [3].

Digital health technologies come in various forms, including mobile platforms, apps, wearables, and Bluetooth- or Wi-Fi–connected devices. Prior research has examined their impact on health outcomes. A study by Vansimaeys et al [4] found that frequent users of multiple digital health felt more empowered and reported stronger patient-doctor relationships. Another randomized controlled trial by Ross and Wing [5] demonstrated that mHealth apps, combined with digital devices, resulted in greater weight loss compared to conventional methods. These findings support the positive role of digital health technologies in disease management [6]. However, despite their promise, many digital devices face challenges with user engagement and adherence [7]. Grounding these tools in behavioral science frameworks and enhancing social validity and user experience are essential [8]. Features such as goal setting, social support, and real-time monitoring may improve motivation and long-term use [9]. Behavioral frameworks such as the health belief model and the unified theory of acceptance and use of technology provide useful lenses to understand what drives users to adopt and sustain digital health practices [10,11].

BMI plays a critical role in disease prevention. Individuals classified as overweight (BMI 24-29.9 kg/m^2^) or obese (BMI≥30 kg/m^2^) are at increased risk of chronic conditions such as diabetes and cardiovascular diseases [12]. While many studies have examined how digital health technologies affect chronic illness management [13-16], few have explored their role in BMI regulation [16-19]. Furthermore, little is known about how individuals without diagnosed conditions, but with varying BMI, use digital tools for health management.

Therefore, understanding how people with different BMI categories engage with digital health technologies is essential for designing effective, tailored interventions. This study aimed to address this gap by (1) investigating the proportion of participants using digital health tools across BMI groups, (2) identifying the types of digital technologies used for health management, and (3) determining the key predictors associated with digital health use. The study analyzed the association between participant characteristics and BMI categories using chi-square tests and regression models while also discussing how usability and design features influence engagement within a medical self-monitoring framework.

## Methods

### Ethical Considerations

This study was approved by the institutional review board committee of National Taiwan University Hospital (registration: 202307210RINC). The study was conducted from January 1, 2024, to April 30, 2024. To protect respondents’ privacy, all survey responses were anonymous, and IP addresses were hidden. This online anonymous survey was determined to fall under the exempt review criteria. As such, no written informed consent was required. Participation was voluntary, and respondents were informed about the purpose of the study at the beginning of the survey. The collected data were used solely for research purposes. Participants were not compensated for their participation in this study.

### Design of the Study

An online anonymous survey was conducted to investigate how people perceive and are aware of their health condition or status, as well as their use of digital devices or mHealth tools (digital health use) for health management. The questionnaire was presented in a fixed sequence determined by logical flow and skip logic, tailored to participants’ responses. Randomization of item order was not applicable due to the structure of conditional branching and mandatory completion of relevant questions. All questions were mandatory unless deemed nonapplicable through skip logic. Respondents were not allowed to skip questions or revise answers once submitted. Trap questions were embedded to identify inattentive or invalid responses. The first module of the questionnaire asked respondents to report their health status or health problems. The second module asked respondents about their health-promoting lifestyle. The third module asked respondents about their self-management behaviors and efficacy in health management. The fourth module focused on the digital health use for monitoring of their health conditions. Data for this study were collected from the survey about the respondents’ demographic characteristics, health perception or awareness, health promotion lifestyle, health motivation, self-efficacy, digital health use, and adherence to mHealth tools.

### Procedure and Participants

The questionnaire designed for the study was hosted on SurveyCake. This platform ensures data integrity, data security, and privacy by using encryption protocols such as Secure Socket Layer to protect data transmission. All collected data were stored on secure servers and are accessible only by authorized personnel. SurveyCake also complies with data protection regulations, ensuring that all personal information is handled in accordance with relevant privacy laws. Participants’ responses were anonymized, and IP addresses were not collected to ensure confidentiality. The link to access the questionnaire was initially disseminated through social networks and emails. Participants were recruited via convenience and snowball sampling methods. The actual scope of dissemination through social media and email forwarding may have reached a broader audience beyond the researcher’s immediate network. The online questionnaire was available on the internet from October 2023 to December 2023 for the pilot run. The target population included Mandarin-speaking adults aged 20 years and older, residing in urban areas. The sample was drawn from a local community where preventive health services are available, but their use may vary across different population groups. For this work, a total of 215 questionnaires were fully completed anonymously. Completion times ranged from 156 to 2292 seconds, with an average of approximately 6.5 (SD 4.2) minutes. Responses with fewer than 90 seconds or more than 1 hour were flagged for review and excluded if deemed invalid. Duplicate responses were not detected directly as IP addresses were not collected, and no cookies were used to prevent multiple entries. Response consistency was checked through logical review and trap questions. Invalid or inconsistent entries were excluded from the analysis. Only fully completed responses were used for the analysis. Therefore, the valid questionnaires were corresponding to 184 respondents (184/215×100%: 85.6%).

### Measures and Variables

Given the online-based approach of the study, a closed-ended questionnaire was used, which included personal factors such as sociodemographic characteristics and health-related information. The questionnaire was developed by referencing validated and reliable instruments to ensure content validity. Expert opinions were also sought during the initial design phase to further refine the questionnaire. The overall design and content were reviewed and approved by the institutional review board prior to conducting the pilot study, which further refined the questionnaire. A pilot study with 35 participants from the target population was conducted to test the feasibility and clarity of the questionnaire, with subsequent modifications made based on feedback. During this process, items with low relevance were removed, and the remaining questions were adjusted to create a new questionnaire tailored to the study’s objectives. This refinement enhanced the internal consistency of the questionnaire, resulting in a Cronbach α exceeding 0.80. The final version included items assessing 5 health-related variables (Table S1 in Multimedia Appendix 1): health awareness, health promotion lifestyle, self-efficacy, health motivation, and digital health use to evaluate respondents’ use of digital health. Participants completed the survey anonymously via an online platform. The questionnaire had been verified by factor analysis and had good reliability (overall Cronbach α=0.90, 66 items; Table S2 in Multimedia Appendix 1). The items in this questionnaire were adapted and simplified from multiple validated instruments, including components of the health belief model and unified theory of acceptance and use of technology–based surveys [10,11].

We surveyed participants about their experience with digital devices, their preferences regarding use frequency, and the parameters they monitor related to their health status. We then examined the percentage distribution of various factors across BMI categories, followed by chi-square analysis. Additionally, the study explored whether respondents had developed a self-management plan, the types of mHealth tools they use, the factors influencing their decision-making, and their satisfaction with and the usability of specific devices or mHealth apps.

### Demographic Characteristics

Demographic characteristics included age (20-29, 30-39, 40-49, 50-59, 60-69, and above 70 years), sex, BMI, educational level (high school or below, college degree, and postgraduate degree), and experience with digital technology.

### Health-Related Variables

#### Health Awareness

Health awareness was measured by assessing respondents’ understanding of the risks associated with obesity, diabetes, and other health conditions. The health awareness score reflects how well individuals recognize the health risks of various diseases and their awareness of the impact of lifestyle choices on overall health [20,21].

#### Health Promotion Lifestyle

A health-promoting lifestyle refers to a set of behaviors that support physical, emotional, and social well-being. In this study, it was assessed using an 11-item simplified scale adapted from the Health Promoting Lifestyle Profile-II [22], covering 6 dimensions: self-actualization, stress management, health responsibility, exercise, interpersonal support, and nutrition [22]. Respondents were asked to rate each statement on a 5-point Likert scale, representing the degree of agreement with each statement. A higher mean score indicates a lifestyle that aligns more closely with an ideal health-promoting lifestyle.

#### Self-Efficacy

Self-efficacy was assessed by measuring respondents’ prior experiences with self-management and their perceived effectiveness in achieving health-related goals [23,24]. Participants rated their confidence in performing specific health management activities using a 5-point Likert scale, with higher scores indicating greater confidence in their ability to successfully manage their health.

#### Health Motivation

This section addresses the factors that influence an individual’s decision to initiate and maintain health-related behaviors, with health motivation defined as the individual’s willingness to invest effort and maintain it in order to achieve health-related goals [25,26]. Motivation includes intrinsic factors, such as the need for disease prevention, as well as extrinsic factors, such as expert advice and social support. This section assesses an individual’s perceived importance of various factors, such as physical health, mental health, disease prevention, and expert advice. Respondents rate the importance of these factors on a scale from 1 to 5, and the total score reflects their overall health motivation.

#### The Digital Health Use

This section evaluated the use of digital health tools, including connected devices, wearable devices, and mobile apps for health management [27-29]. It assessed the types of tools used, the frequency of use, the parameters measured (eg, blood pressure, heart rate, and blood oxygen), and the perceived health improvements associated with these tools [30,31]. For the analysis, this ordinal categorical variable was recoded into a discrete numerical scale, with level 1 representing “less than once per month” and level 5 representing “daily use.” Finally, 3 frequency categories were defined: less than once per week, 1-3 times per week, and more than 3 times per week.

### Statistical Analysis

We used SPSS (version 23.0; IBM Corp) and Python (Python Software Foundation) to conduct all statistical analyses. A *P* value <.05 was considered as statistically significant. The chi-square test or the independent samples 2-tailed *t* test was performed to examine the differences in demographic characteristics and compare each variable of the total population by BMI. The Pearson correlation was analyzed to determine the correlations between the use of digital technology and health-related variables. Moreover, we conducted multiple regression and logistic regression analyses to identify predictors for each health-related variable. Logistic regression was used to analyze the frequency of device use, while multiple regression was applied to analyze the overall variables related to digital health use.

## Results

### Comparison of Sociodemographic Characteristics Across Groups Based on BMI Classification

Table 1 presents the distribution of demographic characteristics, including sex, age, education level, and experience with digital technology, across 3 BMI categories: BMI<24 kg/m^2^, 24≤BMI<29.9 kg/m^2^, and BMI≥30 kg/m^2^. Of the 184 respondents to the valid questionnaires recovered in this study, 71 participants had a BMI<24 kg/m^2^, accounting for 38.6% of the sample; 78 (42.4%) participants had a BMI between 24 and 29.9 kg/m^2^; and 35 (19%) participants had a BMI≥30 kg/m^2^. Chi-square analysis revealed significant differences in sex (*P*<.001) and age (*P*=.02) across the different BMI categories. However, no significant differences were observed in education level or experience with digital technology.

**Table 1:** Demographic characteristics of the sample population by BMI.

Variables				Total (N=184, 100%), n (%)	BMI<24 kg/m^2^ (n=71, 38.6%), n (%)	24≤BMI<29.9 kg/m^2^ (n=78, 42.4%), n (%)		BMI≥30 kg/m^2^ (n=35, 19%), n (%)		*P* value^a^	
**Sex**											<.001^b^
	Male			75 (40.8)	18 (25.4)	34 (43.6)		23 (65.7)			
	Female			109 (59.2)	53 (74.6)	44 (56.4)		12 (34.3)			
**Age (years)**											.02^c^
	20-29			61 (33.2)	35 (49.3)	19 (24.4)		7 (20)			
	30-39			104 (56.5)	29 (40.9)	48 (61.5)		27 (77.1)			
	40-49			10 (5.4)	4 (5.6)	5 (6.4)		1 (2.9)			
	50-59			6 (3.3)	2 (2.8)	4 (5.1)		0 (0)			
	60-69			3 (1.6)	1 (1.4)	2 (2.6)		0 (0)			
**Education**											.38
	High school or below			14 (7.6)	6 (8.5)	4 (5.1)		4 (11.4)			
	College degree			134 (72.8)	54 (76)	54 (69.2)		26 (74.3)			
	Postgraduate degree			36 (19.6)	11 (15.5)	20 (25.7)		5 (14.3)			
**Experience** **with digital technology**											
	**Years of using smartphone**										.41
		>3	179 (97.3)		69 (97.1)	77 (98.7)	33 (94.3)				
		1-3	5 (2.7)		2 (2.9)	1 (1.3)	2 (5.7)				
		None	0 (0)		0 (0)	0 (0)	0 (0)				
	**Weekly frequency of using smartphone**										.45
		>3	183 (99.5)		70 (98.6)	78 (100)	35 (100)				
		1-3	0 (0)		0 (0)	0 (0)	0 (0)				
		<1	1 (0.5)		1 (1.4)	0 (0)	0 (0)				
	**Years of using** **Bluetooth- or Wi-Fi–** **connected device**										.88
		>3	165 (89.7)		62 (87.3)	71 (91)	32 (91.4)				
		1-3	10 (5.4)		5 (7)	3 (3.9)	2 (5.7)				
		None	9 (4.9)		4 (5.7)	4 (5.1)	1 (2.9)				
	**Weekly frequency of using** **Bluetooth- or Wi-Fi–** **connected device**										.15
		>3	155 (84.2)		58 (81.7)	70 (89.7)	27 (77.1)				
		1-3	14 (7.6)		4 (5.6)	5 (6.4)	5 (14.3)				
		<1	15 (8.2)		9 (12.7)	3 (3.9)	3 (8.6)				

^a^Differences based on chi-square test.

^b^*P*<.01.

^c^*P*<.05.

The distribution of personal health information and health perception across different BMI categories (N=184) is shown in Table 2. There were no significant differences in the reported number of personal health problems or family medical history (*P*=.16) or in the types of health-related information accessed in the past 12 months (*P*=.11). Regarding physical activity, most respondents (128/184, 69.4%) engaged in more than 150 minutes per week, with no significant differences between BMI groups. However, chi-square analysis found that the annual frequency of personal medical checkup (*P*=.03) and the self-rating of personal health status (*P*=.02) significantly differed among BMI categories, indicating that participants’ perceptions of their own health varied depending on their BMI. The significance suggested that BMI was associated with how individuals assess their own health. Specifically, as BMI increases, a higher proportion of participants rated their health as “fair to good” (17/35, 48.6% with scores 2-3) or “very poor” (3/35, 8.6% with score 1). Those with a BMI≥30 kg/m^2^ tended to rate their health more negatively compared to those with a lower BMI. However, this did not imply that people with higher BMI were more self-aware about their health, as their annual medical checkup frequency showed an opposite trend (18/35, 51.4% of those with a BMI≥30 kg/m^2^ had fewer than 1 checkup per year). Rather, it may suggest that individuals with higher BMI face more health challenges, which could lead them to perceive their health more negatively. Therefore, it is not necessary that individuals with higher BMI have a stronger sense of self-awareness, but rather that their higher BMI is likely associated with more health issues, which influence how they perceive their overall health.

**Table 2:** Personal health information and health perception of the sample population by BMI (N=184).

Variables			Total, n (%)	BMI<24 kg/m^2^, n (%)		24≤BMI<29.9 kg/m^2^, n (%)		BMI≥30 kg/m^2^, n (%)		*P* value^a^	
**Personal health problems or family medical history**											.16
	1-3	70 (38)		24 (33.8)	35 (44.9)		11 (31.4)				
	>3	4 (2.2)		0 (0)	2 (2.6)		2 (5.7)				
	None	110 (59.8)		47 (66.2)	41 (52.6)		22 (62.9)				
**In 12 months, types of health-related information accessed**											.11
	1-3	90 (48.9)		30 (42.2)	45 (57.7)		15 (42.9)				
	>3	32 (17.4)		10 (14.1)	13 (16.7)		9 (25.7)				
	None	62 (33.7)		31 (43.7)	20 (25.6)		11 (31.4)				
**In 12 months, the attended health education sessions**											.67
	1-3	29 (15.8)		12 (16.9)	10 (12.8)		7 (20)				
	>3	1 (0.5)		0 (0)	1 (1.3)		0 (0)				
	None	154 (83.7)		59 (83.1)	67 (85.9)		28 (80)				
**Weekly physical activity (minutes)**											.86
	<150	56 (30.4)		21 (29.6)	23 (29.5)		12 (34.3)				
	≥150	128 (69.4)		50 (70.4)	55 (70.5)		23 (65.7)				
**Weekly frequency of personal health tracking**											.15
	<1 or none	151 (82.1)		57 (80.3)	62 (79.5)		32 (91.4)				
	1-3	11 (6)		2 (2.8)	7 (9)		2 (5.7)				
	>3	22 (11.9)		12 (16.9)	9 (11.5)		1 (2.9)				
**Annual frequency of personal medical checkup**											.03^b^
	<1 or none	77 (41.8)		27 (38)	32 (41)		18 (51.4)				
	1	101 (54.9)		43 (60.6)	45 (57.7)		13 (37.2)				
	≥2	6 (3.3)		1 (1.4)	1 (1.3)		4 (11.4)				
**The self-rating of personal health status**											.02^b^
	1	4 (2.2)		0 (0)	1 (1.3)		3 (8.6)				
	2-3	79 (43.9)		26 (36.6)	36 (46.2)		17 (48.6)				
	4-5	101 (54.9)		45 (63.4)	41 (52.5)		15 (42.8)				

^a^Differences based on chi-square test.

^b^*P*<.05.

The typology of participants based on their use of multiple digital technologies for their health management was analyzed according to BMI classification (Table 3). Initially, 32.1% (59/184) of respondents reported using Bluetooth- or Wi-Fi–connected devices for their health management. Among them, the use rates for the BMI<24 kg/m^2^, 24≤BMI<29.9 kg/m^2^, and BMI≥30 kg/m^2^ were 33.8% (24/71), 33.3% (26/78), and 25.7% (9/35), respectively, with a *P* value of .67. Furthermore, 49.2% (29/59) of users reported using connected devices less than once per week, 25.4% (15/59) used them 1-3 times per week, and another 25.4% (15/59) used them more than 3 times per week. The BMI<24 kg/m^2^ and 24≤BMI<29.9 kg/m^2^ groups had higher use frequencies, with 50% (12/24 and 13/26) in each group, while the BMI≥30 kg/m^2^ group showed a lower frequency of 44.4% (4/9). However, this difference did not reach statistical significance (*P*=.13). Regarding the number of parameters measured by connected devices, 57.6% (34/59) of users measured 1 or none parameters, 33.9% (20/59) measured 2-4 parameters, and 8.5% (5/59) measured more than 4 parameters. No significant difference was observed among BMI categories (*P*=.36). Additionally, 38.6% (71/184) of respondents reported using wearable devices. The use rates in the BMI categories were 38% (27/71) for BMI<24 kg/m^2^, 39.7% (31/78) for 24≤BMI<29.9 kg/m^2^, and 37.1% (13/35) for BMI≥30 kg/m^2^, with a *P* value of .96. Among the 71 respondents using wearable devices, 56.3% (40/71) used them more than 3 times per week, 21.3% (15/71) used them 1-3 times per week, and 22.5% (16/71) used them less than once per week. Further analysis revealed that the proportion of respondents using wearable devices more than 3 times per week was higher in the BMI<24 kg/m^2^ group (20/27, 74.1%) compared to the other 2 groups (15/31, 48.4% for 24≤BMI<29.9 kg/m^2^ and 5/13, 38.4% for BMI≥30 kg/m^2^), with this difference reaching statistical significance (*P*=.04). Overall, while BMI did not significantly influence the use behavior of digital health devices, respondents with lower BMI demonstrated higher frequency of use of wearable devices.

**Table 3:** The use behavior of digital health devices of the sample population by BMI (N=184).

Variables			Total, n (%)		BMI<24 kg/m^2^, n (%)	24≤BMI<29.9 kg/m^2^, n (%)		BMI≥30 kg/m^2^, n (%)		*P* value^a^	
**The use of connected devices** **for health management**											.67
	Yes	59 (32.1)		24 (33.8)		26 (33.3)	9 (25.7)				
	None	125 (67.9)		47 (66.2)		52 (66.7)	26 (74.3)				
**Weekly frequency of using connected devices** **(for “Yes” respondents)**											.13
	<1	29 (49.2)		12 (50)		13 (50)	4 (44.4)				
	1-3	15 (25.4)		4 (16.7)		10 (38.5)	1 (11.1)				
	>3	15 (25.4)		8 (33.3)		3 (11.5)	4 (44.4)				
**The parameters measured using connected devices (for “Yes” respondents)**											.36
	1 or none	34 (57.6)		14 (58.3)		15 (57.7)	5 (55.6)				
	2-4	20 (33.9)		6 (25)		10 (38.5)	4 (44.4)				
	>4	5 (8.5)		4 (16.7)		1 (3.8)	0 (0)				
**The use of wearable devices for health management**											.96
	Yes	71 (38.6)		27 (38)		31 (39.7)	13 (37.1)				
	None	113 (61.4)		44 (62)		47 (60.3)	22 (62.9)				
**Weekly frequency of using wearable devices (for “Yes” respondents)**											.04^b^
	<1	16 (22.5)		4 (14.8)		6 (19.4)	6 (46.2)				
	1-3	15 (21.3)		3 (11.1)		10 (32.3)	2 (15.4)				
	>3	40 (56.3)		20 (74.1)		15 (48.4)	5 (38.4)				
**The parameters measured using wearable devices (for “Yes” respondents)**											.63
	1 or none	13 (18.3)		4 (14.8)		8 (25.8)	1 (7.7)				
	2-4	51 (71.8)		20 (74.1)		20 (64.5)	11 (84.6)				
	>4	7 (9.9)		3 (11.1)		3 (9.7)	1 (7.7)				
**The use of mobile apps** **for health management**											.61
	Yes	72 (39.1)		34 (47.9)		27 (35.5)	9 (25.7)				
	None	112 (60.9)		37 (52.1)		49 (64.5)	26 (74.3)				
**Weekly frequency of using mobile apps** **(for “Yes” respondents)**											.24
	<1	43 (59.7)		17 (50)		20 (69)	6 (66.7)				
	1-3	21 (29.2)		12 (35.3)		7 (24.1)	2 (22.2)				
	>3	8 (11.1)		5 (14.7)		2 (6.9)	1 (11.1)				

^a^Differences based on chi-square test.

^b^*P*<.05.

Similarly, 39.1% (72/184) of respondents reported using mobile apps for health management. The use rates were 47.9% (34/71) for BMI<24 kg/m^2^, 35.5% (27/78) for 24≤BMI<29.9 kg/m^2^, and 25.7% (9/35) for BMI≥30 kg/m^2^, with no significant difference observed (*P*=.61). Among mobile app users, 59.7% (43/72) used apps less than once per week, 29.2% (21/72) used them 1-3 times per week, and only 11.1% (8/72) used them more than 3 times per week. Although the BMI<24 kg/m^2^ group had a higher proportion of frequent users (5/34, 14.7% with >3 times per week) compared to other groups, this trend did not reach statistical significance (*P*=.24).

While the initial chi-square analysis provided valuable insights into the categorical relationship between BMI categories and the use of digital health devices, it did not account for other potential confounding factors. To gain a more comprehensive understanding of the impact of BMI on digital health device use frequency, we conducted a logistic regression analysis based on all respondents. Table 4 reveals that BMI had no statistically significant impact on the frequency of use for connected devices (BMI coefficient=–0.084; *P*=.76) or wearable devices (BMI coefficient=–0.26; *P*=.25). The finding suggested that BMI did not substantially influence the frequency of use of these devices, as indicated by the low pseudo *R*^2^ and the likelihood ratio (LLR) test *P* values of .76 and .25, respectively. However, a significant negative association was found between BMI and the frequency of mobile app use (BMI coefficient=–0.67; *P*=.02). Specifically, individuals with higher BMI were less likely to use mHealth apps frequently. The model for mobile app use demonstrated a higher pseudo *R*^2^ value of 0.035, and the LLR *P* value was .02, suggesting a moderate explanatory power of the model in predicting mobile app use frequency. By using logistic regression, we were able to evaluate the influence of BMI and identify trends that were not apparent in the initial chi-square analysis, particularly the relationship between higher BMI and reduced frequency of mobile app use.

**Table 4:** Logistic regression analysis of BMI and digital health device use frequency classification results.

Logistic regression model	BMI coefficient (β)	*P* value^a^	Pseudo *R*^2b^	LLR^c^ *P* value
Connected devices	–0.08	.76	0.001	.76
Wearable devices	–0.26	.25	0.006	.25
Mobile apps	–0.67	.02^d^	0.035	.02^d^

^a^Differences based on the regression model.

^b^Pseudo *R*^2^: pseudo coefficient of determination.

^c^LLR: likelihood ratio.

^d^*P*<.05.

### Correlations Between Clusters Based on Health-Related Variables

Table 5 presents the Pearson correlation analysis, which was conducted to examine the relationships between various psychological and behavioral factors, including health awareness, health promotion lifestyle, self-efficacy, health motivation, and digital health use. The results of the analysis provide valuable insights into how these factors are interrelated. Health awareness was positively correlated with self-efficacy (*r*=0.206; *P*=.06), digital health use (*r*=0.333; *P*=.002), and health motivation (*r*=0.525; *P*<.001). These results suggested that individuals with higher health awareness were more likely to demonstrate greater self-efficacy, use digital health technologies more frequently, and exhibit stronger health motivation. Conversely, health promotion lifestyle had a significant negative correlation with self-efficacy (*r*=–0.339; *P*=.002), which may imply that individuals engaged in more health-promoting behaviors may have a diminished sense of personal control over their health outcomes. A similar negative correlation was observed between health promotion lifestyle and health motivation (*r*=–0.214; *P*=.007). This finding suggests that adopting a health-conscious lifestyle does not necessarily lead to higher motivation to improve health. Furthermore, no significant relationship was found between health promotion lifestyle and digital health use (*r*=–0.136; *P*=.03). Self-efficacy correlated positively with both digital health use (*r*=0.359; *P*=.001) and health motivation (*r*=0.469; *P*<.001), indicating that individuals who believe in their ability to manage their health are more likely to engage with digital health tools and pursue health-related goals. Additionally, health motivation displayed a strong positive correlation with digital health use (*r*=0.535; *P*<.001), further emphasizing the role of motivation in driving the adoption of digital health technologies. The Pearson correlation analysis revealed strong associations between self-efficacy, health motivation, and digital health use. The correlation coefficients represent the strength and direction of the relationships between the variables, while the corresponding *P* values indicate the statistical significance of these associations. These results underscore the tendency for individuals who feel confident in managing their health and are highly motivated to improve it to engage more with digital health solutions. In contrast, the health promotion lifestyle showed weaker or negative correlations with other variables, suggesting a more complex interaction that warrants further investigation.

**Table 5:** Pearson correlation between variables (N=184).

			Health awareness	Health promotion lifestyle		Self-efficacy		Digital health use	Health motivation
**Health awareness**									
	*r*	1		–0.149	0.206		0.333		0.525
	*P* value	.001		.04	.06		.001		.001
**Health promotion lifestyle**									
	*r*	–0.149		1	–0.339		–0.136		–0.214
	*P* value	.04		.001	.002		.07		.004
**Self-efficacy**									
	*r*	0.206		–0.339	1		0.359		0.469
	*P* value	.06		.002	.001		.001		.001
**Digital health** **use**									
	*r*	0.333		–0.136	0.359		1		0.535
	*P* value	.001		.07	.001		.001		.001
**Health motivation**									
	*r*	0.525		–0.214	0.469		0.535		1
	*P* value	.001		.004	.001		.001		.001

### Predictors of Digital Health Use

The multiple regression analysis was conducted to examine the predictors of digital health use. This analysis aimed to explore how these factors, including health awareness, health promotion lifestyle, self-efficacy, and health motivation, contributed to the likelihood of engaging with digital health technologies. The results of the regression model are present in Table 6, highlighting the regression coefficients (B), standardized coefficients (β), and the associated *P* values for each predictor. Among the predictors, health awareness (B=0.107; β=0.101; *P*=.32), health promotion lifestyle (B=–0.096; β=–0.054; *P*=.58), and self-efficacy (B=0.122; β=0.098; *P*=.36) were found to have no statistically significant influence on digital health use. This suggested that, by accounting for other variables in the model, these factors did not contribute meaningfully to explaining the variance in digital health use in this particular dataset. In contrast, health motivation (B=0.597; β=0.474; *P*<.001) was found to have a significant positive effect on digital health use, supporting the conclusion that health motivation is a key predictor of digital health use. Data indicated that individuals with higher levels of health motivation were more likely to engage with digital health technologies.

**Table 6:** Multiple regression analysis of overall variables for digital health use^a^.

Predicators	Digital health use		
	B^b^	β^c^	*P* value^d^
Health awareness	0.107	0.101	.32
Health promotion lifestyle	–0.096	–0.054	.58
Self-efficacy	0.122	0.098	.36
Health motivation	0.597	0.474	.001^e^

^a^Adjusted *R*^2^=0.324; *P* value (model) based on *F* test <.001.

^b^B: regression coefficients.

^c^β: standardized coefficients.

^d^2-tailed *t* test for regression coefficients.

^e^*P*<.01.

The overall model’s goodness-of-fit was indicated by the adjusted *R*^2^ value of 0.324, which suggested that approximately 32.4% of the variance in digital health use can be explained by the combination of the predictors included in the model. Furthermore, the overall model was statistically significant, demonstrating that the predictors as a group significantly contribute to explaining digital health use. While health awareness, health promotion lifestyle, and self-efficacy were not significant predictors of digital health use in this analysis, health motivation emerged as a crucial factor influencing engagement with digital health technologies. These findings suggested that interventions aimed at increasing health motivation may be particularly effective in encouraging the use of digital health tools.

### User Engagement With Mobile App Features Across Different BMI Categories

**Figure 1:**
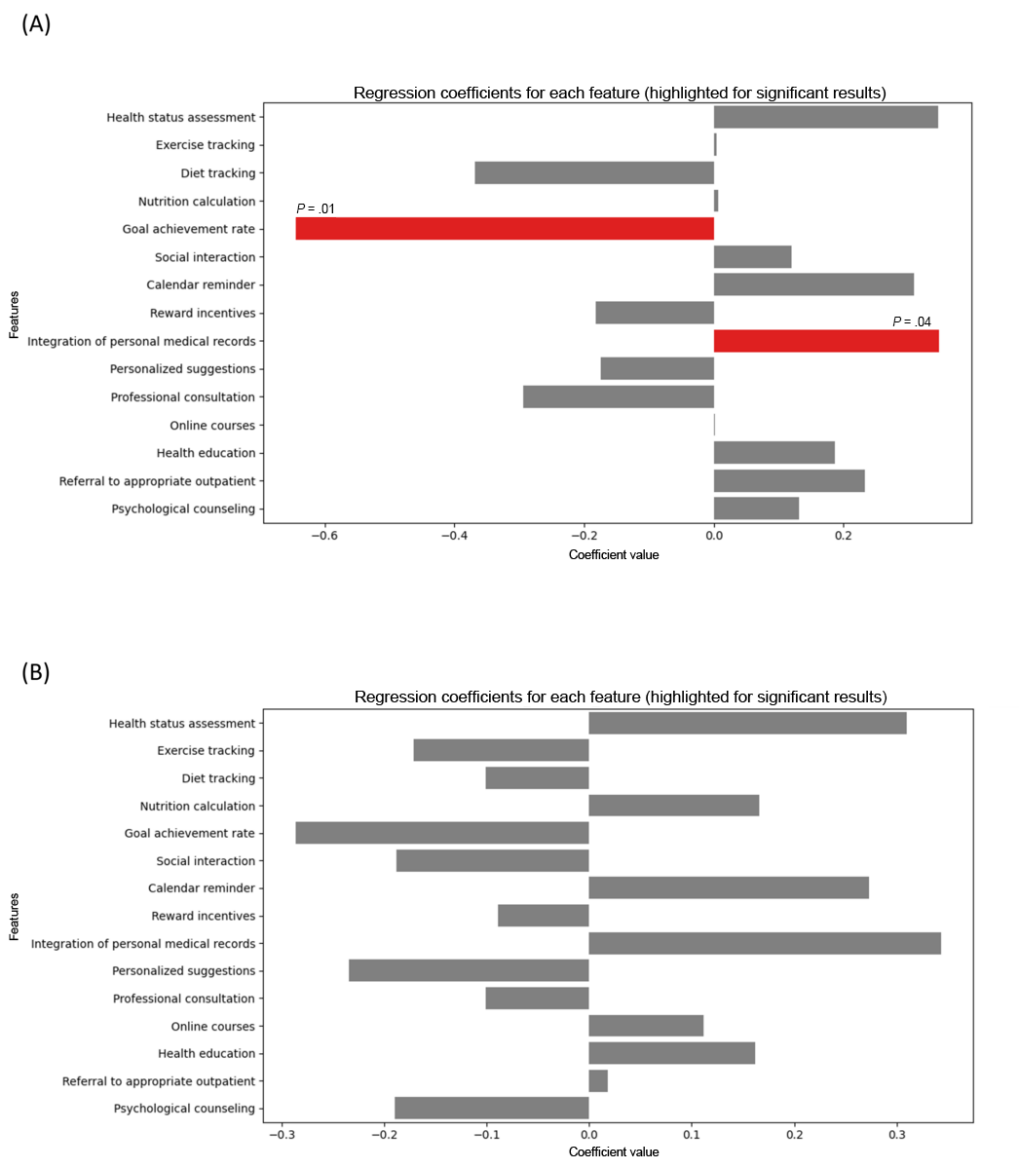
Regression coefficients for (A) 24≤BMI<29.9 kg/m^2^ and (B) BMI≥30 kg/m^2^ with BMI<24 kg/m^2^ as the reference category.

To understand which digital health features (particularly those of mobile apps) might impact user engagement, we further identified which app features were likely to impact individuals with higher BMI (ie, 24≤BMI<29.9 kg/m^2^ and BMI≥30 kg/m^2^). We applied multinomial logistic regression to model the relationship between multiple app features (as independent variables) and BMI categories (as the dependent variable). Using BMI<24 kg/m^2^ as the reference category, the model estimated the differences between the other BMI categories (24≤BMI<29.9 kg/m^2^ and BMI≥30 kg/m^2^) and the BMI<24 kg/m^2^ category. This approach was similar to creating dummy variables for categorical data in regression models, where a categorical variable is represented by binary variables (0 or 1). The coefficient for the reference category (BMI<24 kg/m^2^) was treated as 0, and the coefficients for the other categories (24≤BMI<29.9 kg/m^2^ and BMI≥30 kg/m^2^) reflect their relative differences from this baseline. The results from the multinomial logistic regression model revealed several important aspects about the relationship between app features and BMI categories (Figure 1 and Table S6 in Multimedia Appendix 1). The pseudo *R*^2^ value of 0.128 indicated that approximately 12.8% of the variance in the BMI categories was explained by the model, suggesting a moderate fit. The statistically significant LLR *P* value of .01 further emphasized the relevance of the app features in differentiating the BMI categories. Notably, the feature of integrating personal medical records showed a positive association with individuals in the 24≤BMI<29.9 kg/m^2^ category. For individuals with a BMI≥30 kg/m^2^, while the app features did not show statistically significant differences (*P*=.06), further research will be needed to explore whether individuals in the higher BMI range are more likely to engage with digital health technologies offering similar features.

## Discussion

### Principal Findings

These results indicated that BMI showed significant differences with sex and age, but no significant differences were found in terms of education level and experience with digital technology. Additionally, BMI was negatively correlated with personal health assessment, with participants with higher BMI tending to rate their health more negatively. While BMI did not significantly affect the use of connected and wearable devices for health management, it negatively impacted the frequency of mobile app use, with higher BMI being associated with less frequent use. This analysis not only confirmed the lack of significant relationships between BMI and connected or wearable devices but also highlighted a significant negative association between BMI and the frequency of mobile app use. Additionally, in the multiple regression analysis, health motivation was found to have a significant positive effect on digital health use, whereas health awareness, health promotion lifestyle, and self-efficacy did not significantly influence digital health use. The goodness-of-fit of the overall model indicated that health motivation was an important factor influencing digital health use.

Initially, chi-square analysis was conducted to examine the associations between BMI and device use frequencies, revealing a significant relationship between BMI and wearable device use. However, this analysis did not account for the continuous nature of the data or potential confounding factors. To address these limitations, logistic regression was used to model the likelihood of frequent device use as a function of BMI, providing a more nuanced exploration of the relationship. While no additional confounders were included in the model, the results from logistic regression revealed a significant negative association between BMI and the frequency of mobile app use. This suggested that individuals with higher BMIs may engage less with mHealth apps, possibly due to lifestyle or health-related factors not captured in the initial analysis. The logistic regression also indicated a stronger model fit for mobile app use compared to other devices, highlighting that BMI had a more pronounced effect on mobile app use frequency than on the use of wearable or other connected devices. This difference may be partially explained by the varying levels of user engagement required across device types. Previous research has demonstrated that wearable devices often feature passive data collection and embed behavior change techniques, such as goal setting, self-monitoring, and user feedback [32]. In contrast, mHealth apps generally require more active engagement. For individuals with higher BMI, this may be further complicated by stigma-related discomfort when logging sensitive data (eg, diet or weight). According to the health stigma and discrimination framework proposed by Stangl et al [33], such self-monitoring may intensify awareness of weight-related issues and discourage sustained use. Additionally, apps often incorporate normative comparisons that may heighten psychological burden, whereas wearables operate unobtrusively in the background, minimizing such stressors. These user experience differences may help explain why individuals with higher BMI in our study reported lower app use but no corresponding decline in wearable use.

In this study, we did not find a correlation between the use of digital health tools and health promotion lifestyle, suggesting that digital health technologies may not significantly improve health-related lifestyles or behaviors in this population. In addition, our results indicated that individuals with higher levels of health motivation were more likely to engage with digital health technologies. However, when health motivation was removed from the model, the direct effects of self-efficacy and health awareness became significant predictors of the digital health use (Table S3 in Multimedia Appendix 1). This prompted a deeper investigation into the role of health motivation through mediation analysis, which helps explain the underlying mechanism or pathway by which the independent variables affect the dependent variable. To explore this, a regression analysis was conducted to examine the effects of health awareness and self-efficacy on health motivation. Both factors significantly predicted health motivation, explaining 34.4% of its variance. Further analysis showed that health motivation significantly influenced the digital health use, accounting for 33.7% of the variance in the dependent variable (Tables S4 and S5 in Multimedia Appendix 1). These findings suggested that health motivation partially mediates the relationship between health awareness, self-efficacy, and digital health use.

This mediation was theoretically meaningful because it emphasized that awareness and confidence alone may not automatically lead to digital health engagement without intrinsic motivation to act [23]. Health motivation acted as a bridge that translated an individual’s awareness of health risks and confidence in managing health into actual engagement with digital tools. This mechanism also helped clarify an observation from our data that individuals with higher BMI (≥30 kg/m^2^) showed greater health awareness but lower self-efficacy. Prior literature indicated that while people with obesity may have a high level of awareness regarding their health risks, they often faced complex psychological and behavioral barriers that reduced their confidence to initiate or sustain change [34]. Despite high awareness, individuals with obesity may experience internalized weight stigma and frustration from previous unsuccessful attempts. These emotional burdens, along with feelings of helplessness, could undermine self-efficacy, thereby weakening the link between awareness and proactive behavior [33]. These results suggested that heightened awareness alone was insufficient to drive higher engagement with digital health tools among individuals with higher BMI (≥30 kg/m^2^). To effectively support this population, interventions or app designs should include features that actively cultivate health motivation, the key mediator linking awareness and self-efficacy into action. Features such as personal goal setting, perceived relevance, and meaningful feedback may help reinforce this pathway and encourage sustained health behaviors [35,36].

Therefore, health motivation played a crucial mediating role in driving individuals’ engagement with digital health technologies, suggesting the importance of building both confidence and motivation to encourage proactive health management. mHealth apps and digital devices can serve as a transformative role by empowering individuals to adopt and sustain healthy behaviors. Their interactive features reinforce adherence to health management plans and enhance communication between users and their health care providers [37-39]. This strong predictive power of health motivation may stem from the fact that motivated individuals are more inclined to actively seek out tools that support their health goals. As suggested in previous research, users often perceived digital health technologies as useful tools for achieving behavioral change and self-management [32,35]. Health motivation fostered a proactive mindset, which not only enhanced the perceived usefulness of digital apps but also strengthened the intention to adopt them. Behavioral change is often influenced by perceived outcomes, and digital tools that effectively demonstrate the benefits of specific actions can boost motivation and engagement [40]. Self-efficacy directly influences an individual’s likelihood of engaging in health behaviors and their ability to envision and achieve desired outcomes [41,42]. Higher self-efficacy strengthens the link between intent and action, significantly shaping how individuals adopt and use digital technologies. Consequently, these tools not only enhance health management and disease prevention but also empower individuals to take greater control of their health [43,44]. This empowerment is closely tied to motivation and self-efficacy, both of which are essential for informed decision-making and active participation in health management. The World Health Organization defines empowerment as “a process through which people gain greater control over decisions and actions affecting their health” [45]. This highlights the critical role that self-efficacy and motivation play in helping individuals actively engage in their health management. Furthermore, digital technologies amplify this process by enabling individuals to evaluate and communicate health information more effectively, which requires digital literacy [43]. As a result, the frequency of digital health use also serves as a key indicator of an individual’s self-determination and empowerment.

Our findings indicated that individuals with a higher BMI (≥30 kg/m^2^) demonstrated greater awareness of their health status (eg, lower scores on self-rating of personal health), reflecting relatively stronger health consciousness. However, this heightened awareness did not translate into proactive health behaviors, such as regular annual checkups. The group of BMI≥30 kg/m^2^ exhibited lower confidence in their ability to improve or manage their health, suggesting reduced self-efficacy. While stronger health awareness is evident, the lack of self-efficacy may explain their limited engagement in preventive health measures like regular health checkups. The results align with previous literature and our regression model, both of which highlight health awareness and self-efficacy as key factors influencing health behaviors and motivation [23,32]. Furthermore, studies focused on adolescents diagnosed with overweight or obesity and highlighted that extrinsic motivators like peer support and app-based incentives often led to short-term improvements in BMI [46-48]. This underscored that while digital tools or extrinsic motivators may elicit initial engagement, their long-term effectiveness could be limited. Sustained behavior change, particularly among adults with low self-efficacy, required intrinsic factors such as health motivation, which our findings identified as a critical mediator in digital health adoption.

By fostering a more proactive patient-provider dynamic, mHealth tools encourage shared responsibility and greater engagement in health management [49-51]. Building on this, we further explored which app features are most likely to resonate with individuals with higher BMI (ie, 24≤BMI<29.9 kg/m^2^ and BMI≥30 kg/m^2^). Among these, the integration of personal medical records was positively associated with higher BMI groups, suggesting that this feature may promote greater engagement in health management and potentially lead to improved health outcomes. This association could be attributed to their heightened focus on health management. Individuals with higher BMI often exhibit greater awareness of their health status, which may motivate them to actively track and manage their health information. In this context, the integration of personal medical records could serve as a critical tool, enabling individuals with higher BMI to make more informed health decisions while further reinforcing their health awareness. Conversely, the goal achievement rate exhibited a negative correlation with the likelihood of being classified within the 24≤BMI<29.9 kg/m^2^ category. As the goal achievement rate increased, the probability of individuals being in this BMI range decreased, revealing an inverse relationship between goal achievement and BMI within this group. This suggested that individuals with higher goal achievement rates were less likely to fall into the 24≤BMI<29.9 kg/m^2^ category, potentially reflecting improved health outcomes or more proactive engagement in health behaviors.

These findings are further supported by prior intervention studies, which have demonstrated goal achievement as a key determinant of weight-related outcomes. Burke et al [52] observed that adults who regularly tracked diet and activity through digital platforms achieved clinically significant weight loss (≥5%). Participants who actively set and achieved physical activity goals via apps have shown improved BMI and weight outcomes. The mechanism may function through positive reinforcement and increased self-efficacy, which were associated with sustained behavior change and weight maintenance [53]. In our study, higher goal achievement may reflect more consistent and effective engagement in health-promoting behaviors, thereby supporting BMI levels below 24 kg/m^2^. These insights underscored the importance of building digital health tools that not only enabled goal-setting but also facilitated goal achievement through features like adaptive difficulty or real-time feedback. Such design considerations could be especially beneficial in interventions targeting individuals at risk of overweight or obesity. While the association between goal achievement rate and BMI≥30 kg/m^2^ was not significant, this may be due to the more complex challenges faced by individuals with higher BMIs in terms of health management [52,54]. These individuals often encountered greater barriers, including psychological factors like health stigma and societal pressure, which may undermine their motivation to engage with digital health tools. Additionally, the difficulty in setting and achieving realistic goals may lead to frustration and disengagement, further contributing to the lack of a significant association. Overall, our findings demonstrated that app features may influence individuals across different BMI categories, potentially enhancing engagement with digital health technologies, particularly among those with higher BMI. However, further research is needed to explore the relationship between psychological and motivational factors and the underlying mechanisms driving these patterns.

### Limitations

While this study provided valuable insights, several limitations should be noted. The sample size of 184 participants and the focus on a regional population may limit the generalizability of the findings, particularly concerning BMI subgroups and regional or ethnic factors. The participants were recruited from an urban area, a region where preventive health services were accessible but not uniformly used. Health management in this setting was often influenced by cultural emphasis on body image, familial expectations, and access to publicly funded health care services. These factors may shape both health awareness and behavioral engagement in ways that differ from populations in Western countries or other regions. Additionally, the study relied on self-reported data for health assessments and digital health use, which may be subject to biases such as social desirability or recall bias. These biases could have led to overreporting of healthy behaviors or engagement with digital tools, potentially affecting the observed associations. To address this, future research could incorporate objective measures (eg, app use logs or wearable device data) to validate self-reports. Moreover, expanding the sample size and including a more diverse population would strengthen the findings and improve the external validity of the results. Future studies could adopt more rigorous sampling strategies, including stratified or probability sampling to ensure better representation across BMI categories, regions, and socioeconomic backgrounds. Longitudinal studies will be needed to assess the directionality of the relationships between BMI, health behaviors, and digital health technology use. This study focused on psychological factors such as health awareness, self-efficacy, and motivation. Other unmeasured confounding variables such as socioeconomic status, physical activity levels, or comorbid conditions could also influence the relationships observed between BMI and health-related behaviors. These factors were not controlled in the current design and may partially explain the variability in digital health engagement and health behavior adoption. Finally, while this study focused on wearables and mobile apps and their features, future research should explore other digital health tools, such as telemedicine platforms, to investigate their role in health management across different BMI categories. These tools often provide additional functionalities, such as real-time virtual consultations, personalized feedback, and long-term health monitoring, which may be particularly valuable for individuals with higher BMI who require continuous support or tailored interventions. Moreover, to better understand the underlying psychological or emotional factors influencing digital health engagement, qualitative research such as in-depth interviews could provide richer insights into user experiences, perceived barriers, and motivational drivers.

### Conclusions

The results of this study indicate a relationship between BMI, health motivation, and the use of digital health technologies, particularly in the use of mHealth apps. Although BMI did not significantly affect the frequency of digital health device use, a higher BMI was associated with a lower frequency of mobile app use, suggesting that individuals with higher BMI are less likely to use mHealth apps frequently. This implies that individuals with higher BMI may exhibit differences in health motivation, which in turn impacts their frequency of digital health tool use. Furthermore, in the multiple regression analysis, health motivation was found to have a significant positive influence on digital health use. This suggests that health motivation plays a crucial role in influencing the use of digital health technologies, with individuals exhibiting higher health motivation showing greater engagement. Communication strategies should also consider users’ preferences by offering flexible notification options, such as message, email, or in-app settings, allowing individuals to tailor the frequency and type of communication they receive. By emphasizing the integration of personalized health data, health care providers could potentially increase user engagement, particularly among individuals with higher BMIs, by providing more tailored and relevant health insights. This could improve their frequency of use and ultimately encourage healthier behaviors. Additionally, improving the accessibility and usability of mHealth tools may help foster greater engagement across different BMI groups.

## Data Availability

The datasets used and analyzed during this study are available from the corresponding author on reasonable request.

## Appendix

Multimedia Appendix 1 Supplementary tables summarizing measurement tools, reliability and validity analyses, and regression models used in this study.

## Abbreviations

LLR: likelihood ratio
mHealth: mobile health
