# Supplementary material for: Health Motivation as a Predictor of mHealth Engagement Across BMI: Cross-Sectional Survey
Source: JMIR Mhealth Uhealth. 2025 Dec 1;13:e71625. doi: 10.2196/71625 (PMC12706447; doi:10.2196/71625)
Supplement: Multimedia Appendix 1 [file mhealth_v13i1e71625_app1.docx]

**Table S1** lists the questionnaire items used in this study to assess five health-related variables.

| Variables | Content | Item Numbers |
| --- | --- | --- |
| Health Awareness | Health awareness was measured by assessing respondents' understanding of the risks associated with obesity, diabetes, and other health conditions. The health awareness score reflects how well individuals recognize the health risks of various diseases and their awareness of the impact of lifestyle choices on overall health. | 11 items |
| Health Promotion Lifestyle | Concepts including self-actualization, stress management, health responsibility, exercise, interpersonal support and nutrition | 11 items |
| Self‐efficacy | Previous experience on self-management and effectiveness, goal achievement rate | 8 items |
| Health Motivation | We measured health motivation by focusing on individuals' perceived importance of various factors, including both intrinsic factors, such as the desire for disease prevention, and extrinsic factors, such as expert advice and social support. | 8 items |
| The Digital Health Use | The types of mobile applications, wearable devices and connected devices, the usage frequency, the parameters measured, the improvement extent and importance of the app functions | 28 items |

**Table S2** presents the reliability and validity analysis of the study, which included 66 items with an overall Cronbach’s alpha of 0.90.

|  | Cronbach’s α |
| --- | --- |
| Health Awareness | 0.91 |
| Health Promotion Lifestyle | 0.85 |
| Self‐efficacy | 0.84 |
| Health Motivation | 0.88 |
| The Digital Health Use | 0.95 |

**Table S3** shows the results of the multiple regression analysis of overall variables influencing digital health use, excluding health motivation.

| Predicators | Digital Health Use | | |
| --- | --- | --- | --- |
|  | B | β | *p* value ^c^ |
| Health Awareness | 0.283 | 0.268 | .010* |
| Health promotion lifestyle | -0.207 | -0.117 | .27 |
| Self‐efficacy | 0.329 | 0.264 | .017* |
| Adjusted R^2^ | 0.183 | | |
| *p* value^d^ (Model) | .001** | | |

^c^ Differences based on t-test, ***p* < .01, **p* < .05

^d^ Differences based on F-test, ***p* < .01, **p* < .05

B: Regression Coefficients, β Standardized Coefficients, Adjusted R^2^: Adjusted Coefficient of Determination

**Table S4** shows the multiple regression analysis examining the relationship between health awareness, self-efficacy, and health motivation.

| Predicators | Health Motivation | | |
| --- | --- | --- | --- |
|  | B | β | *p* value ^c^ |
| Health Awareness | 0.315 | 1.233 | 0.001** |
| Self‐efficacy | 0.410 | 0.253 | 0.001** |
| Adjusted R^2^ | 0.344 | | |
| *p* value^d^ (Model) | 0.001** | | |

^c^ Differences based on t-test, ***p* < .01, **p* < .05

^d^ Differences based on F-test, ***p* < .01, **p* < .05

B: Regression Coefficients, β Standardized Coefficients, Adjusted R^2^: Adjusted Coefficient of Determination

**Table S5** shows the multiple regression analysis exploring the effect of health motivation on digital health use.

| Predicators | Digital Health Use | | |
| --- | --- | --- | --- |
|  | B | β | *p* value ^c^ |
| Health Motivation | 0.698 | 0.341 | 0.001** |
| Adjusted R^2^ | 0.337 | | |
| *p* value^d^ (Model) | 0.001** | | |

^c^ Differences based on t-test, ***p* < .01, **p* < .05

^d^ Differences based on F-test, ***p* < .01, **p* < .05

B: Regression Coefficients, β Standardized Coefficients, Adjusted R^2^: Adjusted Coefficient of Determination

**Table S6** summarizes the results from the multinomial logistic regression (MNLogit) analysis of BMI categories and mobile app features.

| Multinomial Logistic Regression Model | BMI Range | BMI Coefficient (β) | *p* value^b^ | Pseudo R^2^ | LLR  *p* value |
| --- | --- | --- | --- | --- | --- |
| Intercept  (BMI <24) | BMI <24 | - | - | 0.128 | 0.014* |
| Health status assessment | 24≤BMI <29.9 | 0.347 | .06 |  |  |
|  | BMI ≥30 | 0.310 | .15 |  |  |
| Exercise tracking | 24≤BMI <29.9 | 0.005 | .98 |  |  |
|  | BMI ≥30 | -0.171 | .42 |  |  |
| Diet tracking | 24≤BMI <29.9 | -0.369 | .05 |  |  |
|  | BMI ≥30 | -0.101 | .64 |  |  |
| Nutrition calculation | 24≤BMI <29.9 | 0.007 | .97 |  |  |
|  | BMI ≥30 | 0.166 | .41 |  |  |
| Goal achievement rate | 24≤BMI <29.9 | -0.645 | .001** |  |  |
|  | BMI ≥30 | -0.287 | .11 |  |  |
| Social interaction | 24≤BMI <29.9 | 0.121 | .49 |  |  |
|  | BMI ≥30 | -0.188 | .38 |  |  |
| Calendar reminder | 24≤BMI <29.9 | 0.309 | .07 |  |  |
|  | BMI ≥30 | 0.273 | .15 |  |  |
| Reward incentives | 24≤BMI <29.9 | -0.182 | .278 |  |  |
|  | BMI ≥30 | -0.089 | .65 |  |  |
| Integration of personal medical records | 24≤BMI <29.9 | 0.348 | .038* |  |  |
|  | BMI ≥30 | 0.343 | .056 |  |  |
| Personalized suggestions | 24≤BMI <29.9 | -0.174 | .28 |  |  |
|  | BMI ≥30 | -0.235 | .20 |  |  |
| Professional consultation | 24≤BMI <29.9 | -0.293 | .18 |  |  |
|  | BMI ≥30 | -0.101 | .62 |  |  |
| Online courses | 24≤BMI <29.9 | 0.002 | .99 |  |  |
|  | BMI ≥30 | 0.112 | .59 |  |  |
| Health education | 24≤BMI <29.9 | 0.187 | .26 |  |  |
|  | BMI ≥30 | 0.162 | .38 |  |  |
| Referral to appropriate outpatient | 24≤BMI <29.9 | 0.233 | .21 |  |  |
|  | BMI ≥30 | 0.018 | .93 |  |  |
| Psychological counseling | 24≤BMI <29.9 | 0.132 | .46 |  |  |
|  | BMI ≥30 | -0.190 | .37 |  |  |

b Differences based on regression model, ***p* < .01, **p* < .05

Pseudo R^2^: Pseudo coefficient of determination, LLR *p* value: Likelihood Ratio Test *p* value.
